# The Varieties of Uterine Neoplasms and Their Relative Frequency

**Published:** 1899-12

**Authors:** W. Roger Williams


### THE VARIETIES OF UTERINE NEOPLASMS AND THEIR RELATIVE FREQUENCY.

BY

W. Roger Williams, F.R.C.S. Eng.

Uterine tumours are of great interest to the surgeon and pathologist, as well for the frequency of their occurrence as for the important surgical procedures now often undertaken for their removal. It was very different half a century ago, when but little was known of the pathology of these tumours, and their surgical treatment was limited to the occasional removal of specimens that projected into the vagina.

In this essay, I propose briefly to set forth some results of a statistical investigation of uterine neoplasms; and then to make passing reference to the bearing of the facts thus revealed on the question of pathogenesis.

The great frequency of uterine tumours is shown by the following data:—

Of 13,824 patients of both sexes, with primary neoplasms, consecutively under treatment at four large London hospitals, I have ascertained that 2,649 were of uterine origin, or 19.2

per cent.; while next in order of frequency came the mammæ with 17.5 per cent.; then the skin with 9.4 per cent.; and far removed from the last, the stomach with only 2.6 per cent.

Similarly of 13,971 neoplasms analysed by Gurlt, the patients being under treatment at the chief Vienna hospitals, 4,115 originated from the uterus, or 29 per cent.; next in order came the skin with 12 per cent., the mammæ with 11 per cent., and the stomach with 8 per cent.

With regard to the sex of the patients in my list, 9,227 were females: in 28.7 per cent. of these women the uterus was the organ affected; the mammæ in 26 per cent., the ovaries in 8.7 per cent., and the stomach in only 1.4 per cent.

In striking contrast with the foregoing, I find that of 4,597 neoplasms in males only 25, or .5 per cent. were of the mammæ.

Of my 13,824 neoplasms, 7,297 were cancers: of the latter 4,628 occurred in persons of the female sex, the uterus being the seat of origin in 1,571, or in 34 per cent., the mammæ in 40.3 per cent., the skin in 4.1 per cent., and the stomach in 2·8 per cent.

Of Gurlt's 13,971 neoplasms, 9,898 were cancers: 7,020 of the latter occurred in persons of the female sex, the uterus being affected in 3,449, or in 49 per cent., the mammæ in 20 per cent., and the stomach in 7 per cent.

For comparison with the above clinical data, I append some of the results deducible from the chief mortality statistics.

From the Registrar-General's analysis of the cancer mortality of England and Wales for the year 1897, I find that among females the uterus, etc., was the seat of the disease in 23.5 per cent., the mammæ in 15.5 per cent., the stomach in 13.3 per cent., and the liver in 13.2 per cent.: similar returns have been published for the years 1888 and 1868; they show the following percentages: uterus 34·7, mammæ 21.2, and stomach 10.9.

From the reports of the Registrar for Ireland, for the years 1887–89, it appears that among women the stomach was the part affected in 22.4 per cent., mammæ in 21.5 per cent., and uterus in 14.1 per cent.

The Frankfort-on-Main mortality returns for the 30 years, 1860–89, show that the uterus was the seat of the disease in 27.5 per cent. of the female cancer mortality, the stomach in 18.5 per cent., and the mammæ in 11.3 per cent.

It will be gathered from the foregoing that these mortality statistics differ from the clinical data, chiefly in that they indicate greater frequency of the disease in the stomach, etc.

Schroeder's analysis of 19,666 cases of cancer in women shows that 33.3 per cent. were uterine; and of 8,746 similar cases tabulated by Simpson, 34.3 per cent. were of the uterus.

Of the 9,227 females with neoplasms in my list, in 2,649 (28.7 per cent.) the uterus was the part affected. In these cases the relative frequency of the occurrence of the different varieties of uterine tumour is shown by the subjoined table:

ANALYSIS OF 2,649 CONSECUTIVE CASES OF UTERINE NEOPLASMS.

| | |
|---|---|
| Cancer ... ... ... ... ... | 1,571 |
| Sarcoma ... ... ... ... ... | 2 |
| Myoma ... ... ... ... ... | 883 |
| Polypus (non-myomatous) ... | 191 |
| Cystoma ... ... ... ... ... | 2 |
| Total ... ... ... ... | 2,649 |

Of Gurlt's 4,115 uterine neoplasms, 3,449 were cancers, 8 sarcomas, 481 myomas, 175 polypoid pseudoplasms, and 2 were papillomas.

Throughout the organism in general, malignant neoplasms occur, in females, with greater relative frequency than non-malignant ones, the ratio being—according to my estimate—55 per cent. of the former to 45 per cent. of the latter. In the uterus the proportionate numbers are 59.38 per cent. of the former to 40.62 per cent. of the latter.

In order to show the relative frequency of the chief uterine neoplastic manifestations in comparison with those of the female organism in general, and with those of the female mammæ, ovaries, skin and stomach (in females), I have compiled the subjoined table:—

TABLE SHOWING THE RELATIVE FREQUENCY OF FEMALE NEOPLASMS IN GENERAL, AS COMPARED WITH UTERINE, MAMMARY, CUTANEOUS, GASTRIC, AND OVARIAN NEOPLASMS.

| KIND OF NEOPLASM. | Female Neoplasms in General per cent. | Uterine Neoplasms per cent. | Female Breast Neoplasms per cent. | Skin Neoplasms in Females per cent. | Gastric Neoplasms in Females per cent. | Ovarian Neoplasms per cent. |
|---|---|---|---|---|---|---|
| Cancer ... ... | 48.7 | 59.30 | 77.7 | 34.1 | 100 | 3.36 |
| Sarcoma... ... | 6.3 | .08 | 3.9 | 1.8 | nil. | 2.98 |
| Non-Malignant Neoplasms | 33.4 | 40.54 | 15.7 | 29.1 | nil. | .12 |
| Cysts ... ... | 11.6 | .08 | 2.7 | 35.0 | nil. | 93.54 |

This shows that the proneness of the different organs to evolve the various neoplasms is extraordinarily variable. Thus, while in some organs certain neoplasms hardly ever arise, these same organs nevertheless often originate other neoplasms, although the latter are of the rarest occurrence in yet other organs.

Thus, although the proneness of the uterus to originate cancer, as compared with its proneness to originate other neoplasms, is above the average for females in general, yet it is much surpassed in this respect by the stomach and mammæ; the liability of the skin is, however, much less, while that of the ovaries is quite insignificant.

On the other hand, so great is the relative frequency of cancer of the stomach, as compared with its liability to other neoplasms and cysts, that for practical purposes the very existence of these latter may be ignored.

Although the liability of all these organs to sarcoma is much below the average, yet *inter se* the relative frequency of its occurrence presents considerable variations: in the uterus and stomach, for instance, sarcoma is remarkably rare; whereas in the female breast, ovaries, and skin it is relatively not so very uncommon.

The most striking feature in the neoplastic pathogeny of the uterus, however, is its great relative proneness to non-malignant growths; with this the almost complete immunity of the stomach and ovaries from such growths contrasts markedly.

Again, while the relative proneness of the uterus and stomach to originate cysts is infinitesimal, yet tumours of this kind arise in the ovaries with such preponderating frequency as to reduce the ratio of all other ovarian tumours to insignificant proportions.

In every part of the body, where neoplasms arise, we meet with similar phenomena.

These extraordinary differences in morbid proclivity are among the most remarkable facts in the whole range of neoplastic pathogeny; and no doubt the solution of the problem of the origin of neoplasms concentres in them.

It seems to me impossible to account for such vagaries, otherwise than as the result of biological peculiarities inherent to the various tissues of the affected parts. No doubt in every such locality there must be corresponding morphological changes, although the microscope has hitherto failed adequately to reveal them. In this connection some recent observations of Ribbert are of importance. He has shown that cancer is most prone to arise from epithelia in which active mitotic changes are normally always present, or in which such changes manifest themselves under certain conditions (as in the mammæ); whereas in organs whose epithelia seldom exhibit mitoses, such as the salivary glands, lachrymal glands, thyroid, thymus, male mammæ, etc., cancers seldom arise. These observations give direct anatomical support to the doctrine I have long advocated on other grounds; *viz.*, that cancers are most prone to arise in localities where cells still capable of growth and development most abound.

From the fact that no part of the body—not even the mamma — undergoes so many remarkable post-embryonic developmental changes as the uterus—which moreover also possesses unique reparative powers—we may infer that it is unusually rich in cells, still retaining much of their embryonic capabilities. The behaviour of the uterus, as compared with the tube, under the stimulus of pregnancy, strikingly illustrates these remarks. When a fertilised ovum lodges in the uterine cavity, the walls of the latter grow so rapidly, that they readily adapt themselves to the requirements of the nascent embryo; but when the ovum is arrested in the Fallopian tube and develops there—although at first the tubal structures grow so as to accommodate it—yet, as the embryo augments, the increase of these structures fails to keep pace with it, so that the tube is eventually ruptured.

It is probably owing to inherent peculiarities of this kind, that the uterus is so much more prone to originate neoplasms than other parts of the body. In like manner, the great proclivity of certain regions of the uterus to similar outbreaks, and the comparative immunity of other regions, may probably be explained. At any rate, it is evident that the influence of locality in determining the genesis, structure, and qualities of uterine neoplasms is very great.

---

